# Enteric Glia Cells Attenuate Cytomix-Induced Intestinal Epithelial Barrier Breakdown

**DOI:** 10.1371/journal.pone.0069042

**Published:** 2013-07-01

**Authors:** Gerald A. Cheadle, Todd W. Costantini, Nicole Lopez, Vishal Bansal, Brian P. Eliceiri, Raul Coimbra

**Affiliations:** Division of Trauma, Surgical Critical Care, and Burns, Department of Surgery, University of California San Diego Health Sciences, San Diego, California, United States of America; Charité-University Medicine Berlin, Germany

## Abstract

**Background:**

Intestinal barrier failure may lead to systemic inflammation and distant organ injury in patients following severe injury. Enteric glia cells (EGCs) have been shown to play an important role in maintaining gut barrier integrity through secretion of S-Nitrosoglutathione (GSNO). We have recently shown than Vagal Nerve Stimulation (VNS) increases EGC activation, which was associated with improved gut barrier integrity. Thus, we sought to further study the mechanism by which EGCs prevent intestinal barrier breakdown utilizing an *in vitro* model. We postulated that EGCs, through the secretion of GSNO, would improve intestinal barrier function through improved expression and localization of intestinal tight junction proteins.

**Methods:**

Epithelial cells were co-cultured with EGCs or incubated with GSNO and exposed to Cytomix (TNF-α, INF-γ, IL-1β) for 24 hours. Barrier function was assessed by permeability to 4kDa FITC-Dextran. Changes in tight junction proteins ZO-1, occludin, and phospho-MLC (P-MLC) were assessed by immunohistochemistry and immunoblot.

**Key Results:**

Co-culture of Cytomix-stimulated epithelial monolayers with EGCs prevented increases in permeability and improved expression and localization of occludin, ZO-1, and P-MLC. Further, treatment of epithelial monolayers with GSNO also prevented Cytomix-induced increases in permeability and exhibited a similar improvement in expression and localization of occludin, ZO-1, and P-MLC.

**Conclusions & Inferences:**

The addition of EGCs, or their secreted mediator GSNO, prevents epithelial barrier failure after injury and improved expression of tight junction proteins. Thus, therapies that increase EGC activation, such as VNS, may be a novel strategy to limit barrier failure in patients following severe injury.

## Introduction

The intestinal barrier is comprised of epithelial cells that are linked together by tight junctions, which are comprised of the proteins zonula occludens-1 (ZO-1) and occludin, which form the tight junction barrier in the paracellular space [1]. Connected to these proteins is the actin cytoskeletal ring and myosin light chain (MLC), which help to further stabilize the tight junction [2,3]. Together, these proteins help to maintain the gut barrier against the external environment of the intestinal lumen. Breakdown of the intestinal tight junction results in increased intestinal permeability and gut inflammation. This can lead to the spread of gut-derived inflammatory mediators to the mesenteric lymph, contributing to the development of systemic inflammation [4,5]. On a cellular level, gut barrier breakdown is associated with decreased expression and altered localization of the tight junction proteins occludin [6] and ZO-1 [7]. Additionally, phosphorylation of MLC causes actin cytoskeletal contraction, increasing tight junction breakdown [2,3,8]. Severe injury models [9,10] and chronic diseases of intestinal inflammation [11,12] have been associated with increases in intestinal permeability resulting from tight junction disruption.

Because gut barrier breakdown is associated with systemic inflammation, we postulate that therapies designed to prevent intestinal inflammation may have clinical utility in the treatment of medical conditions associated with gut barrier failure. Recent evidence suggests Vagus nerve stimulation (VNS) may modulate intestinal barrier integrity [13], through improved expression of occludin [6] and ZO-1 [14] and a reduction in phosphorylated MLC (P-MLC) [15]. However, the signaling mechanism that links the central nervous system and the intestinal epithelium remains elusive.

One potential signaling pathway may lie in the well-established connections between the Vagus nerve and the Enteric Nervous System (ENS). The largest cell population in the ENS are the Enteric Glia cells (EGCs), which are identified by the unique marker Glial Fibrillary Acidic Protein (GFAP) [16]. Recently, it has been demonstrated that EGCs actively receive and propagate signals, both to and from nearby enteric neurons and the intestinal epithelium [16–18]. Additionally, VNS was shown to increase intestinal GFAP expression, a common marker of EGC activation, suggesting a connection between the Vagus nerve and EGCs [15]. Thus, EGCs may be an ideal candidate cell type to transmit the anti-inflammatory effects of VNS to the gut epithelium.

The potential for EGCs to mediate VNS-induced gut protection are supported by studies showing that EGCs play a prominent role in maintaining proper intestinal epithelial barrier integrity. Genetic ablation of EGCs results in intestinal barrier failure and is associated with increased intestinal inflammation [18,19]. Subsequent studies have shown that the barrier-inducing effects of EGCs are mediated through the secretion of S-nitrosoglutathione (GSNO), which improves expression and localization of tight junction proteins [19].

Thus, with evidence suggesting that EGCs may link VNS to the intestinal epithelium, this project seeks to further investigate the effects of EGCs and GSNO on epithelial barrier function using an *in vitro* co-culture model with EGCs and well-established epithelial cell lines. We hypothesized that the addition of EGCs, or their secreted mediator GSNO, would prevent epithelial barrier failure after exposure to an inflammatory stimulus through improved expression of tight junction proteins.

## Materials and Methods

### Cell lines

Caco-2 human intestinal epithelial cells, EGCs, and Madin Darby Canine Kidney (MDCK) epithelial cells were obtained from American Type Culture Collection (ATCC, Manasas, VA, USA). All three cell lines were grown at 37°C in a 5% CO_2_ humidified atmosphere. Cells were grown in Dulbecco’s Modified Eagle Medium (DMEM) with high glucose (Gibco, Carlsbad, CA, USA) supplemented with 10% FBS (Gibco), penicillin G (10,000 U mL^-1^, Gibco), and streptomycin (10,000 µg mL^-1^, Gibco). MDCKs were supplemented with L-Glutamine (2 mM, Gibco), Sodium Pyruvate (1 mM, Gibco), and 1% nonessential amino acids (Gibco).

### Co-culture Model and Immunostimulation

Caco-2 cells (80,000 cells well^-1^) or MDCK cells (30,000 cells well^-1^) were seeded onto permeable filters with 0.4 µm pore size in 12-well Transwell bicameral chambers (Corning Inc., Corning, New York, USA). EGCs (30,000 cells well^-1^) were seeded onto the basal well of the 12-well Transwell dish. Media was changed daily throughout the co-culture process. After the incubation period, cells were subject to permeability assays, immunoblot, or immunofluorescence protocols.

For the Caco-2 co-culture, cells were seeded on day 0 and allowed to grow for two days alone in DMEM. EGCs were plated on day 1 and allowed to grow for one day alone in DMEM. Cells were then co-cultured on day 2, and grown together for another 3 days. On day 3, Caco-2 cells were incubated in Enterocyte Differentiation media (BD Bioscience, Bedford, MO, USA) to help induce Caco-2 differentiation. On day 4, cells were placed in serum-free media and incubated with either PBS or Cytomix (TNF-α (10 ng mL^-1^; Sigma), IFN-γ (10 ng mL^-1^; Pierce, Rockford, IL, USA), and IL-1β (10 ng mL^-1^; Sigma)) for 24 hours at 37°C in a 5% CO_2_ humidified atmosphere. For GSNO studies (50 µM, Sigma), incubation began in selected Caco-2 samples on the same day as co-culture and was added to the basal well. GSNO was replenished daily with media changes in the same time frame as other Caco-2 samples were co-cultured with EGCs. To block the effects of GSNO, the nitric oxide synthase inhibitor *NG*-nitro-L-arginine methylester (100µmol/L) was incubated with EGCs prior to assessing permeability.

For the MDCK co-culture, MDCK cells were seeded on day 0 and allowed to grow for two days alone in DMEM. EGCs were plated on day 1 and allowed to grow for one day alone in DMEM. Cells were then co-cultured on day 2 or incubated with GSNO as described and allowed to grow together for two days. On day 4, cells were placed in serum-free media and incubated with either PBS or Cytomix for 24 hours at 37°C in a 5% CO_2_ humidified atmosphere.

### Permeability Assay

To assess monolayer barrier function, an *in vitro* permeability assay was performed. After the 24 hour Cytomix incubation period, 200 µL of 4kDa FITC-Dextran (10 mg mL^-1^; Sigma) in PBS was added to the Transwell insert, or the apical side of the monolayer. After 4 hours of incubation, 100 µL aliquots of DMEM were obtained from the basal chamber. Fluorescence was measured in a fluorescence spectrometer (SpectraMax, Molecular Devices, Sunnyvale, CA, USA) and compared with a standard curve of known FITC-Dextran concentrations diluted in PBS and DMEM.

### Immunofluorescence

After the co-culture procedure, media was removed from the Transwells and fixed in 3.7% paraformaldehyde (Electron Microscopy Sciences, Hatfield, PA, USA) diluted in PBS. Cells were fixed for 15 minutes. Cells were then washed with PBS for 2 minutes. After washing, the cells were blocked in a 3% BSA solution for 1 hour. Cells were washed and incubated overnight in primary antibodies against ZO-1 (1:500, Invitrogen, Camarillo, CA, USA), occludin (1:500, Invitrogen), and Phosphorylated Myosin Light Chain (P-MLC; 1:500, Santa Cruz Biotechnology, Santa Cruz, CA, USA) in 1% BSA solution. After washing, cells were incubated in secondary antibody, Alexa Fluor 488 (1:1000, Invitrogen), and 4,6-diamidino-2-phenylindole (DAPI; 1:200, Sigma) in 1% BSA for one hour. Cells were washed, followed by removal of the Transwell membranes, containing the cells, from the inserts. The membranes were immersed in SlowFade Gold (Invitrogen), placed on glass slides, and covered with glass cover slips. Slides were allowed to cure overnight in the dark. Images were then viewed with an Olympus Fluoview laser scanning confocal microscope (Olympus, Melville, NY, USA) at 60x magnification with exposure-matched settings (Advanced Software V 1.6, Olympus, Center Valley, PA, USA).

### Immunoblot

At the end of the culture procedure, cells were washed with PBS and placed in 0.25% Trypsin-EDTA (Gibco). Once cells were in suspension, they were removed from the Transwells, put into Eppendorf tubes, and placed in a centrifuge at 14,000 rpm for 5 minutes. Supernatant was removed and the tubes placed on ice. 250 µL of 4% Sodium dodecyl sulfate (SDS) in PBS lysis buffer and 5 µL of Protease Inhibitor (Pierce) were added to the tubes, followed by sonication to lyse the cellular pellet. Total protein samples were placed at -80°C for long-term storage.

Total protein concentration of the lysed samples was determined by the bicinchoninic acid (BCA) protein assay using the microplate procedure (Pierce). Samples containing 10 µg of protein were placed into SDS sample buffer and boiled for 5 minutes. Proteins were separated by SDS-Polyacrylamide gel electrophoresis using 8-16% tris-glycine polyacrylamide gradient gel and transferred to nitrocellulose membranes (Invitrogen). Membranes were washed with tris-buffered saline/Tween 20 (TBST) and then blocked with 5% BSA in TBST for 1 hour. Membranes were then incubated in primary antibodies specific for occludin (1:500, Santa Cruz Biotechnology) or Beta Actin (1:500, Cell Signaling, Danvers, MA, USA) overnight at 4°C in 5% BSA in TBST. Membranes were washed and then incubated with horseradish peroxidase-linked anti-rabbit IgG (1:2000, Cell Signaling) in 5% BSA in TBST for 1 hour at room temperature. After washing, the Pierce Supersignal West Pico Chemiluminescent Kit was applied to the membranes for antibody detection through the Xenogen IVIS Lumina (Caliper Life Science, Hopkinton, MA, USA) imaging system. Mean pixel density was determined using the UN-SCAN-IT Gel Digitizing software (Silk Scientific, Orem, UT, USA). Band densities were compared to the Beta Actin band densities in each lane as a loading control. Data is expressed as the relative band density compared to control for each experiment.

### Statistical Analysis

All values are expressed as mean ± standard error of the mean (SEM). Statistical significance was determined using analysis of variance (ANOVA) with Student-Newman-Keuls correction. A *p*-value < 0.05 was considered statistically significant.

## Results

### EGCs attenuate Cytomix-induced epithelial monolayer permeability

**Figure 1:**
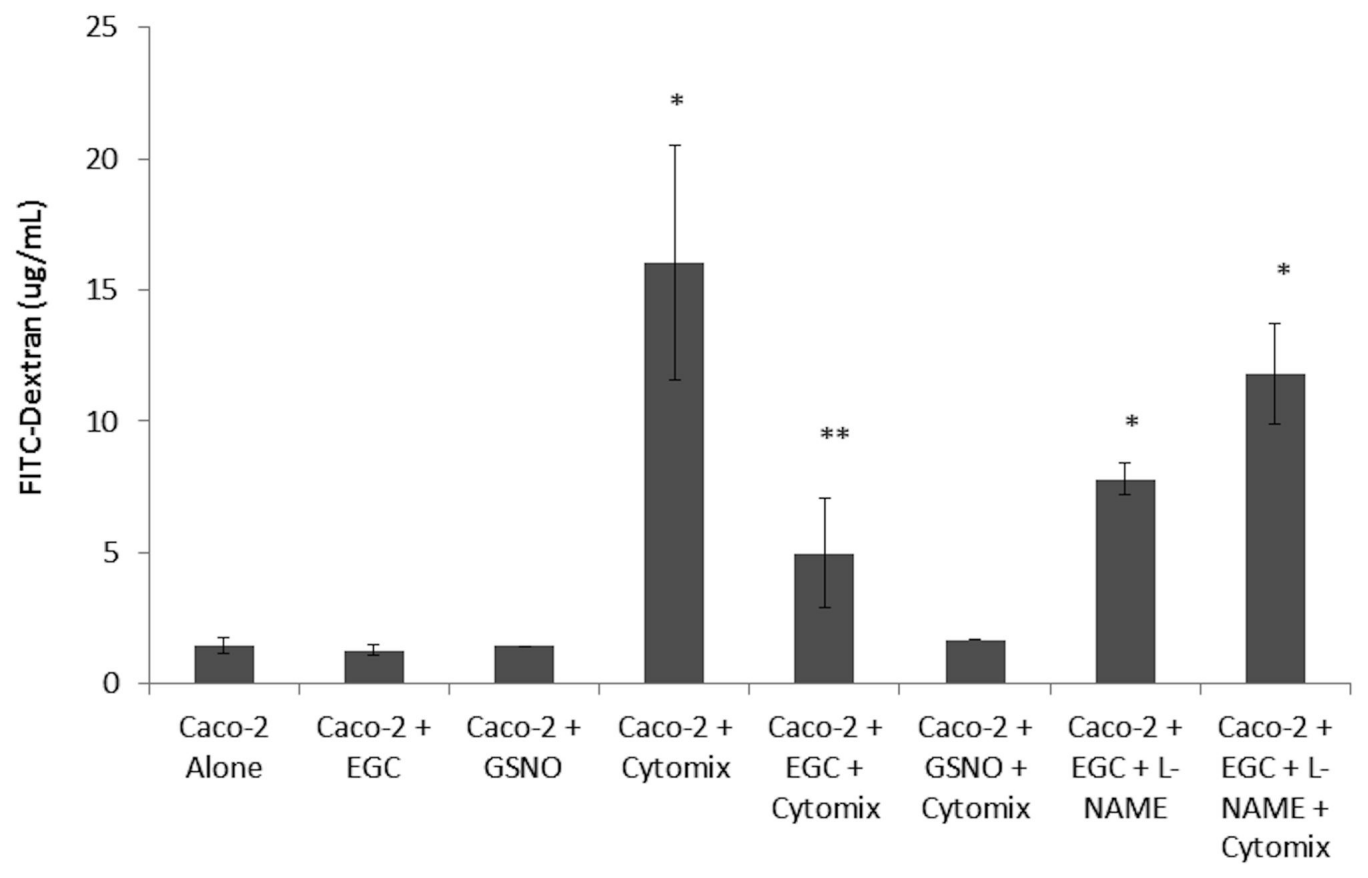
EGCs and GSNO attenuate Cytomix-induced monolayer permeability in Caco-2 cells. Caco-2 cells were grown in either the presence or absence of EGCs or GSNO and incubated with Cytomix (TNF-α, IFN-γ, IL-1β) or PBS for 24 hours. L-NAME was used to block GSNO activity from EGCs. Caco-2 monolayer permeability to 4kDa FITC-Dextran was measured (n ≥ 4 samples per group). Cytomix-stimulation results in an increase in monolayer permeability, indicating barrier dysfunction. The presence of either EGCs or GSNO significantly reduces permeability levels. *p < 0.05 versus the controls Alone, + EGC, + GSNO ; **p < 0.01 versus + Cytomix, and +EGC +L-NAME +Cytomix.

To assess Caco-2 barrier integrity, an *in vitro* permeability assay was performed using FITC-dextran (Figure 1). Caco-2 monolayers stimulated with Cytomix demonstrated an increase in paracellular permeability. Co-culturing EGCs with Caco-2 epithelial monolayers prevented Cytomix-induced barrier failure, with permeability restored to control levels. Further, if the stimulated epithelial cells were incubated with GSNO, the secreted product of EGCs thought to propagate their barrier-inducing effects, there is a similar reduction in permeability. Blocking nitric oxide synthase using L-NAME abrogated the barrier protective effects of EGCs suggesting that the barrier protective effects of EGCs are mediated in part by GSNO.

### EGCs improve localization of the tight junction protein ZO-1 in Cytomix-stimulated epithelial monolayers

**Figure 2:**
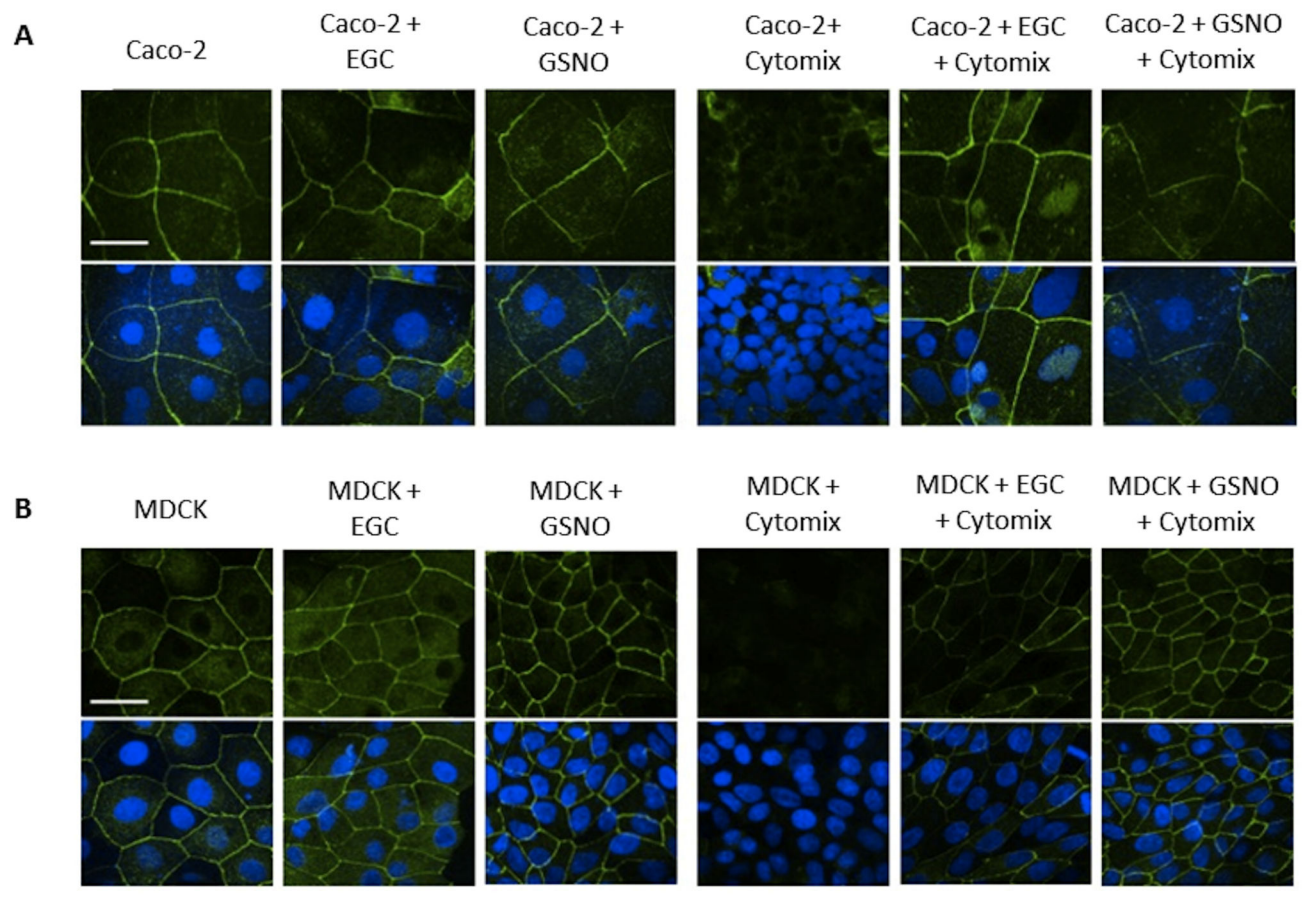
EGCs and GSNO improve localization of ZO-1 in Caco-2 and MDCK cells. Epithelial cells were grown in either the presence or absence of EGCs or GSNO and incubated with Cytomix (TNF-α, IFN-γ, IL-1β) for PBS for 24 hours. *A*: Caco-2 monolayers stained with anti-ZO-1 antibodies (green) and DAPI (blue) and imaged through confocal microscopy. Cytomix-stimulated monolayers have an altered localization of ZO-1 away from the cell surface compared to controls, indicating tight junction disruption. Stimulated cells co-cultured with EGCs or incubated with GSNO exhibited a restoration of ZO-1 localization at the cell surface. *B*: MDCK cells stained with anti-ZO-1 antibodies (green) and DAPI (blue) and imaged through confocal microscopy. Cytomix-stimulated monolayers have an altered localization of ZO-1 indicating tight junction disruption. Stimulated cells co-cultured with EGCs or incubated with GSNO demonstrated normal distribution of ZO-1. Images are of 60x magnification and exposure matched. Bar = 30µm.

After determining the effects of Cytomix, EGCs, and GSNO on epithelial permeability, we sought to determine if these effects were due to changes in tight junction protein expression. We first examined ZO-1 localization in Caco-2 (Figure 2A) and MDCK (Figure 2B) monolayers using confocal microscopy. Staining of ZO-1 in control samples of both epithelial cell lines demonstrated localization at areas of cell contact, forming a smooth, continuous, outline of cellular borders. Stimulation of epithelial cell monolayers with Cytomix altered ZO-1 localization away from the cell surface, indicating tight junction disruption. However, co-culture of Cytomix-stimulated monolayers with EGCs restored normal ZO-1 localization at areas of cell contact. Further, incubation of stimulated-epithelial cells with the EGC-secreted product, GSNO, alone also restored normal ZO-1 cellular localization, replicating the effect of EGCs.

### EGCs improve occludin expression in Cytomix-stimulated epithelial monolayers

**Figure 3:**
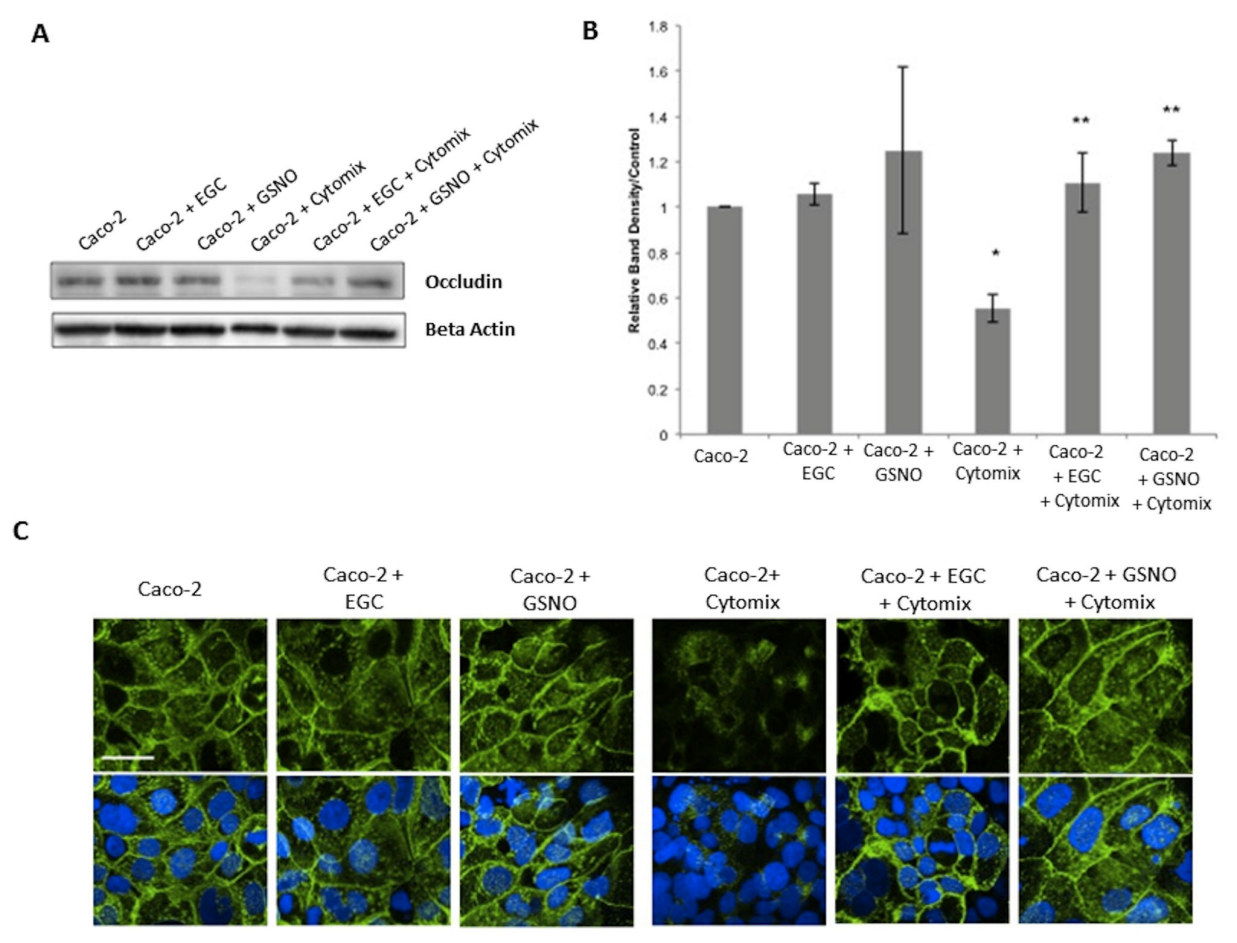
EGCs and GSNO improve expression and localization of occludin in Caco-2 cells. *A*–*B*: Caco-2 occludin immunoblot and relative band densities. Cytomix-stimulation decreased occludin expression compared to controls. Co-culture of stimulated-cells with EGCs or incubation with GSNO prevented the Cytomix-induced loss of occludin expression. *C*: Caco-2 monolayers stained with anti-occludin antibodies (green) and DAPI (blue) and imaged through confocal microscopy. Cytomix-stimulated monolayers have an altered localization of occludin away from the cell surface compared to controls, indicating tight junction disruption. Stimulated cells co-cultured with EGCs or incubated with GSNO exhibited a restoration of occludin localization at the cell surface. Images are of 60x magnification and exposure matched. Bar = 30µm. *p < 0.05 versus Caco-2 cells alone, Caco-2 + EGC, Caco-2 + GSNO; **p < 0.05 versus Caco-2 + Cytomix using analysis of variance (ANOVA).

**Figure 4:**
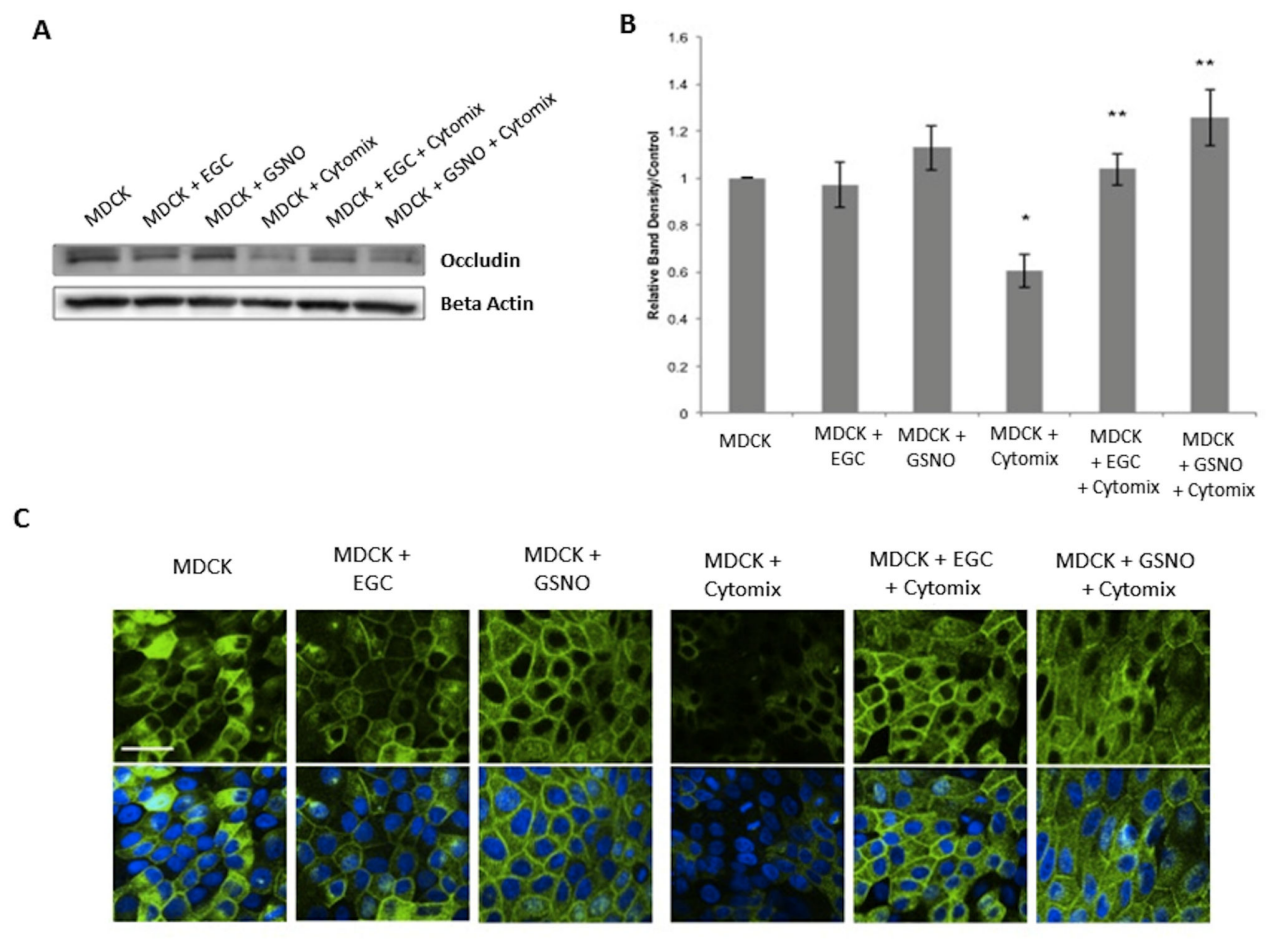
EGCs and GSNO improve expression and localization of occludin in MDCK cells. *A*–*B*: MDCK occludin immunoblot and relative band densities. Cytomix-stimulation decreased occludin expression compared to controls. Co-culture of stimulated-cells with EGCs or incubation with GSNO prevented the Cytomix-induced loss of occludin expression. *C*: Caco-2 monolayers stained with anti-occludin antibodies (green) and DAPI (blue) and imaged through confocal microscopy. Cytomix-stimulated monolayers have an altered localization of occludin away from the cell surface compared to controls, indicating tight junction disruption. Stimulated cells co-cultured with EGCs or incubated with GSNO exhibited a restoration of occludin localization at the cell surface. Images are of 60x magnification and exposure matched. Bar = 30µm. *p < 0.05 versus MDCK cells alone, MDCK + EGC, MDCK + GSNO ; **p < 0.05 versus MDCK + Cytomix using analysis of variance (ANOVA).

Next, we examined the tight junction protein occludin in both epithelial cell lines by both immunoblot and immunohistochemistry. Stimulation of monolayers with Cytomix caused a 45% ± 6.0 and a 41% ± 7.2 decrease in occludin expression as compared with controls in Caco-2 and MDCK cells, respectively (Figures 3A-B, 4A-B). Co-culture with EGCs prevented Cytomix-induced loss of occludin expression. Further, incubation with the secreted product, GSNO, alone also prevented the Cytomix-induced loss of occludin expression.

To determine the localization of occludin in the epithelial cells, confocal images were examined in both epithelial cell lines (Figures 3C, 4C). Control samples showed a pattern of proper occludin organization and localization at the cell surface. Cytomix-stimulated epithelial monolayers showed an altered localization of occludin away from the cell surface, suggesting tight junction disruption. However, co-culture of stimulated epithelial cells with EGCs restored occludin localization to the cell surface. Further, epithelial cells incubated with GSNO alone exhibited a similar restoration of normal occludin localization.

### EGCs decrease phosphorylated Myosin Light Chain (P-MLC) expression in Cytomix-stimulated epithelial monolayers

**Figure 5:**
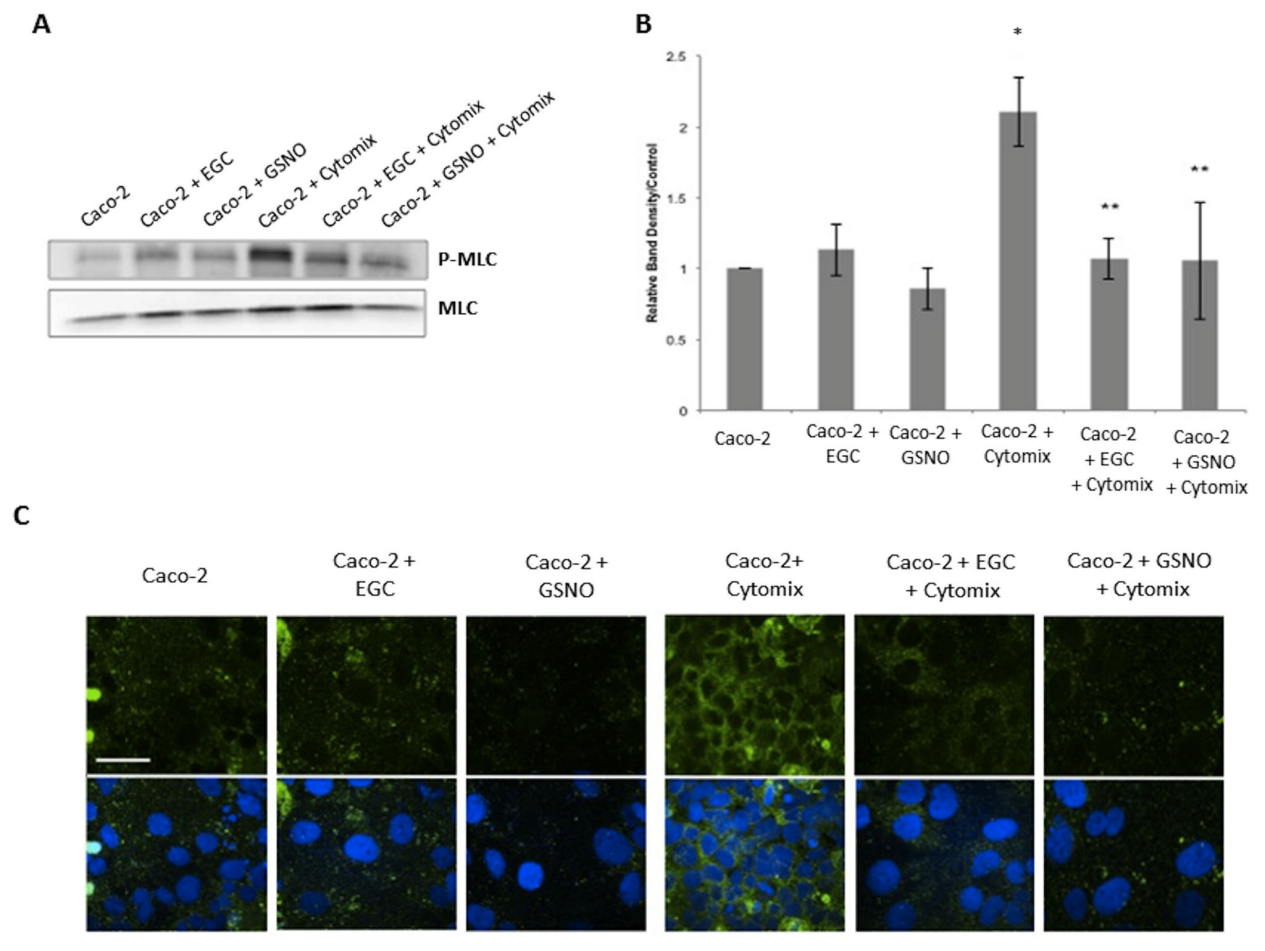
EGCs and GSNO reduce expression of P-MLC in Caco-2 cells. *A*–*B*: Caco-2 P-MLC immunoblot and relative band densities. Cytomix-stimulation increased P-MLC expression compared to controls. Co-culture of stimulated-cells with EGCs or incubation with GSNO prevented the Cytomix-induced increase in P-MLC expression. *C*: Caco-2 monolayers stained with anti-P-MLC antibodies (green) and DAPI (blue) and imaged through confocal microscopy. Cytomix-stimulated monolayers have increased levels of P-MLC compared to controls, indicating tight junction disruption. Co-culture of Cytomix-stimulated cells with EGCs or incubation with GSNO restores normal levels of P-MLC. Images are of 60x magnification and exposure matched. Bar = 30µm. *p < 0.05 versus Caco-2 cells alone, Caco-2 + EGC, Caco-2 + GSNO; **p < 0.05 versus Caco-2 + Cytomix using analysis of variance (ANOVA).

**Figure 6:**
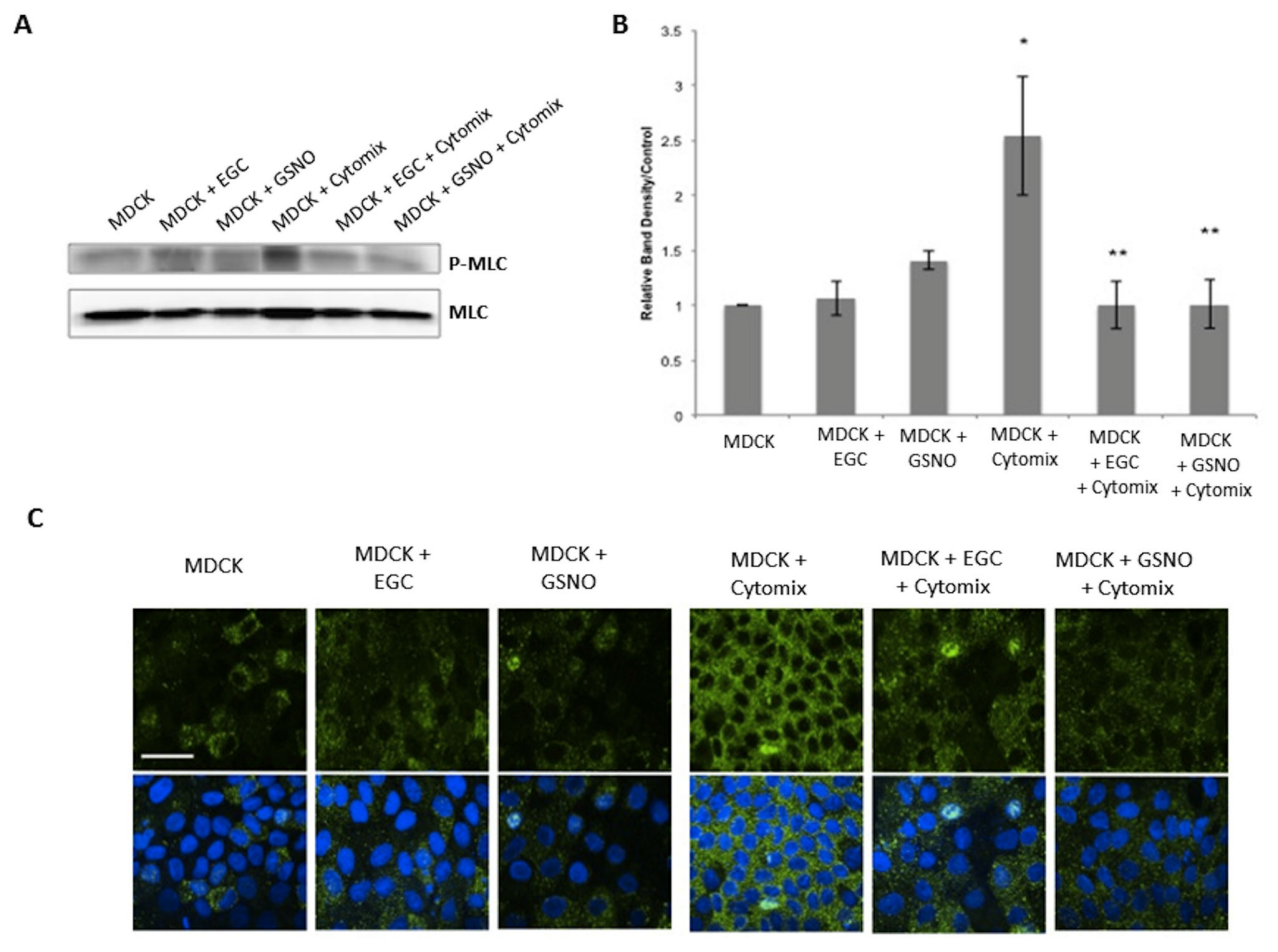
EGCs and GSNO reduce expression of P-MLC in MDCK cells. *A*–*B*: MDCK P-MLC immunoblot and relative band densities. Cytomix-stimulation increased P-MLC expression compared to controls. Co-culture of stimulated-cells with EGCs or incubation with GSNO prevented the Cytomix-induced increase in P-MLC expression. *C*: MDCK monolayers stained with anti-P-MLC antibodies (green) and DAPI (blue) and imaged through confocal microscopy. Cytomix-stimulated monolayers have increased levels of P-MLC compared to controls, indicating tight junction disruption. Co-culture of Cytomix-stimulated cells with EGCs or incubation with GSNO restores normal levels of P-MLC. Images are of 60x magnification and exposure matched. Bar = 30µm. *p < 0.05 versus MDCK cells alone, MDCK + EGC, MDCK + GSNO; **p < 0.05 versus MDCK + Cytomix using analysis of variance (ANOVA).

It has been shown that immunostimulation of intestinal epithelial cells results in an increase in Myosin Light Chain Kinase (MLCK) activity [2,3,8]. Increased expression of MLCK causes an increase in phosphorylation of MLC, which is associated with actin cytoskeletal contraction, tight junction disruption, and epithelial barrier breakdown [8]. Thus, we decided to examine P-MLC by both immunoblot and immunohistochemistry in both epithelial cell lines. Stimulation of monolayers with Cytomix caused a 2-fold and a 2.5-fold increase in P-MLC expression as compared with controls in Caco-2 and MDCK cells, respectively (Figures 5A-B, 6A-B). Co-culture with EGCs prevented Cytomix-induced increase in P-MLC expression. Further, incubation with the secreted product, GSNO, alone also prevented the Cytomix-induced increase in P-MLC expression.

To further visualize P-MLC levels, confocal images were examined in both epithelial cell lines. Control samples established low, basal levels of P-MLC (Figures 5C, 6C), which is expected in normal conditions. Cytomix-stimulated epithelial monolayers had increased P-MLC levels as shown by increased fluorescence staining, which is associated with tight junction disruption. Epithelial cells that were co-cultured with EGCs, however, demonstrated reduced staining for P-MLC, with levels comparable to control. Further, epithelial cells incubated with GSNO alone demonstrated a similar reduction in P-MLC levels, indicating barrier protection.

## Discussion

The intestinal barrier plays a critical role in preventing gut inflammation in patients following severe injury. Breakdown of the gut barrier is associated with increases in systemic inflammation, distant organ injury, and death [4,6,7,14,20]. Studies have shown that modulation of intestinal epithelial tight junction proteins, through VNS, may be a potential strategy for attenuating gut barrier failure after injury [5,6,13–15]. In this study, we explore the signaling pathway by which VNS maintains gut barrier integrity after injury, specifically focusing on the role of EGCs. We first showed the importance of EGCs and one of their secreted products, GSNO, in preventing epithelial barrier breakdown in response to pro-inflammatory cytokines in an *in vitro* co-culture model with EGCs and epithelial cells. The importance of GSNO was further confirmed by incubating EGCs with the NOS inhibitor L-NAME to inhibit the effects of GSNO. L-NAME prevented EGC-mediated barrier protection in monolayers exposed to Cytomix, supporting data from prior studies demonstrating that blocking GSNO limits the barrier-protective effects of EGCs [19]. EGCs and GSNO both appear to modulate their effects on intestinal epithelial cells through improved expression and localization of tight junction proteins ZO-1, occludin, and P-MLC.

This *in vitro* co-culture model affords us the ability to completely isolate intestinal epithelial cells and EGCs to examine potential signaling mechanisms, which are responsible for modulation of epithelial barrier function. However, the challenge arises on how to properly simulate injury and barrier breakdown that is comparable to *in vivo* models. We chose the pro-inflammatory cytokines TNF-α, IFN-γ, and IL-1β, collectively referred to as Cytomix, as they have been well validated in multiple studies to induce epithelial barrier breakdown *in vitro* and simulate the effects of inflammation-induced barrier failure [21–24]. Additionally, while MDCKs are not intestinal epithelial cells, they have widely recognized as a good epithelial cell model for use as corroborating evidence in intestinal barrier studies [19,25]. Thus, they were used in this study primarily to corroborate the data found in the Caco-2 cell line.

Studies have shown that gut-derived inflammatory mediators are carried into the mesenteric lymph following injury, which can contribute to the development of distant organ injury [4]. Thus, breakdown of intestinal tight junction proteins, causing increased permeability, is associated with a systemic inflammatory response and significant morbidity and mortality in injured patients. In these experiments, we showed that injury-induced altered localization of the proteins ZO-1 and occludin, as well as increases in P-MLC levels, are associated with increased epithelial permeability *in vitro*. Other studies have shown similar correlations between intestinal permeability and tight junction breakdown *in vivo*. In a murine model of traumatic brain injury (TBI) [20] or severe burn injury [6,7,14], increases in intestinal barrier failure, as evidenced by increased paracellular permeability and histologic gut injury, were associated with decreased expression or altered localization of both occludin and ZO-1.

Therapies aimed at protecting intestinal barrier integrity may have important clinical relevance as a means to decrease the gut barrier failure after injury. While the focus in our lab has remained on the ability of VNS to signal to EGCs, several studies have shown that enteric neurons may also be involved in maintaining intestinal epithelial barrier integrity. Studies by both Conlin, et al. and Neunlist, et al. have revealed the importance of the enteric neuron-secreted mediator, vasoactive intestinal peptide (VIP), which reduces intestinal barrier permeability and improves tight junction protein expression [26,27]. Given this data and the well-established connections between the Vagus nerve and the ENS, it is conceivable that VNS may also be directly innervating enteric neurons to secrete barrier-inducing mediators such as VIP. Examining the effects of the Vagus nerve on enteric neurons are points of further study in our laboratory to help further define the intestinal barrier-protective effects of VNS.

However, our laboratory has focused on the ability of VNS to activate EGCs, altering the intestinal inflammatory response by modulating local gut injury [15,28]. While this suggests a connection between the Vagus nerve and EGCs, the signaling mechanism remains unknown. However, the study by MacEachern, et al. suggests that nicotinic cholinergic agonists mediate signaling between enteric neurons and EGCs. After having identified the presence of the α3-nicotinic cholinergic receptor on the surface of EGCs, they found that stimulation of these receptors with multiple cholinergic agonists results in the release of nitric oxide (NO) from EGCs, which plays an important role in proper epithelial ion transport [29]. Additionally, studies suggest that the Vagus nerve propagates several of its anti-inflammatory signals through nicotinic cholinergic mechanisms. Borovikova, et al. demonstrated that cholinergic agonists such as acetylcholine, nicotine, and muscarine all were able to replicate the anti-inflammatory effects of VNS [30–33]. Finally, studies in our laboratory revealed that stimulation of the α-7 nicotinic cholinergic receptor by nicotine also replicated the intestinal barrier protective effects of VNS [28]. Thus, mounting evidence suggests EGCs are a logical candidate to propagate the anti-inflammatory effects of VNS in the intestine through a nicotinic cholinergic signaling mechanism.

Therefore, we sought to examine the effects of EGCs on injured intestinal epithelial cells. Our results indicate that EGCs reduce Cytomix-induced epithelial permeability by improving tight junction protein expression and localization. Many other studies identify similar roles for EGCs in maintaining intestinal homeostasis [25,34,35]. In concert with their barrier-inducing effects, EGCs have also been shown to play a prominent role in epithelial mucosal restitution [34]. Microarray studies revealed that EGCs appear to modulate intestinal epithelial cell gene expression by increasing expression of cell adhesion, differentiation, and motility genes, while subsequently decreasing proliferation gene expression [35]. The study by Neunlist, et al. was able to identify that EGCs secreted Transforming Growth Factor-β (TGF-β), which was responsible for the observed anti-proliferative effects. This study and others have shown that the primary method of communication between EGCs and the intestinal epithelial cells is through secreted mediators [16–18,29]. In addition to TGF-β, Savidge, et al. identified that GSNO is the barrier-inducing factor secreted by EGCs [19]. While we report that GSNO appears to replicate the barrier-inducing effects of EGCs in our model, other soluble mediators may be of importance and should be points of further study.

A major finding in this study is that GSNO replicates the barrier-inducing effects of EGCs. We report that GSNO improves the expression and localization of the intestinal tight junction proteins ZO-1, occludin, and P-MLC. The importance of GSNO in proper intestinal function has been shown in other studies [15,36]. It has been reported that GSNO replicated the protective effects of EGCs in preventing barrier disruption in *in vitro* models of *Shigella flexneri* infection, a clinically relevant enteroinvasive pathogen [36]. Further, in our lab, we have shown that in severe injury models, intraperitoneal GSNO treatment was able to replicate the protective effects of VNS in a model of severe burn injury [15]. Thus, it appears that GSNO is a potent barrier-inducing molecule when administered to epithelial cells.

While this is certainly an important finding, subsequent studies should attempt to examine the molecular signaling of GSNO that results in improved barrier function. GSNO is a potent nitric oxide (NO) donor, which can function to S-nitrosylate proteins, a post-translational modification. Studies have suggested that proteins can be S-nitrosylated on cysteine residues, which can dramatically alter their function [37–39]. GSNO may alter the gut inflammatory response through its ability to alter Nuclear Factor κB (NF-κB) inflammatory signaling, where it has been shown that S-nitrosylation of inhibitory κB kinase (IKK) by GSNO inhibits its ability to phosphorylate inhibitor of κB (IκB). Further, S-nitrosylation of NF-κB has been shown to inhibit its ability to bind DNA to initiate transcription of pro-inflammatory mediators [39]. Altering NF-κB inflammatory signaling may have important effects on gut barrier failure, as binding of the NF-κB transcriptional factor in intestinal epithelial cells is associated with tight junction disruption [40]. In our laboratory, we observed a similar mechanism, in which burn injury results in increased phosphorylation of IκB and NF-κB, which is associated with increased intestinal tight junction breakdown [8]. With the suggested connections between VNS, GSNO, and improved tight junction protein expression in this study, it is conceivable that GSNO may act to inhibit the NF-κB pathway through protein S-nitrosylation.

Taken together, our data suggest that EGCs, potentially through their ability to secrete GSNO, improves barrier integrity by preventing inflammation-induced changes in intestinal tight junction protein expression. The signaling mechanism defined in this project has set the groundwork for developing targeted drug therapies for intestinal barrier breakdown. Such therapies may be a novel treatment strategy aimed at limiting the systemic inflammatory response and late deaths in patients following severe injury.
